# An Optimum Specimen Geometry for Equibiaxial Experimental Tests of Reinforced Magnetorheological Elastomers with Iron Micro- and Nanoparticles

**DOI:** 10.3390/nano7090254

**Published:** 2017-09-03

**Authors:** Luis Manuel Palacios-Pineda, Imperio Anel Perales-Martínez, Mario Regino Moreno-Guerra, Alex Elías-Zúñiga

**Affiliations:** 1 Escuela de Ingenieria y Ciencias, Tecnologico de Monterrey, Monterrey 64849, Mexico; palacios@itpachuca.edu.mx (L.M.P.-P.); anel.perales@itesm.mx (I.A.P.-M.); marioregino@hotmail.com (M.R.M.-G.); 2 División de Estudios de Posgrado e Investigación, Instituto Tecnológico de Pachuca, Pachuca 42082, Mexico

**Keywords:** biaxial tests, cruciform specimen, Mullin’s effect, nanoparticles

## Abstract

The aim of this paper focused on obtaining the optimum cruciform geometry of reinforced magnetorheological elastomers (MRE) to perform homogeneous equibiaxial deformation tests, by using optimization algorithms and Finite Element Method (FEM) simulations. To validate the proposed specimen geometry, a digital image correlation (DIC) system was used to compare experimental result measurements with respect to those of FEM simulations. Moreover, and based on the optimum cruciform geometry, specimens produced from MRE reinforced with carbonyl-iron microparticles or iron nanoparticles were subjected to equibiaxial loading and unloading cycles to examine their Mullin’s effect and their residual strain deformations.

## 1. Introduction

The addition of materials of different nature, size, and morphology to neat polymers leads to new reinforced polymeric composites with physical and mechanical properties that need to be determined, since during their manufacturing processes or service conditions [1,2,3] these could be subjected to three-dimensional deformation states that require a good understanding of their mechanical properties to predict their qualitative and quantitative behavior [4,5]. In this sense, the common practice of using tensile testing to predict the material’s response is insufficient to account for anisotropic effects that could arise in reinforced polymeric materials. Therefore, the experimental data obtained from these tensile tests do not provide sufficient information to appropriately identify constitutive equations to describe the material behavior under a variety of complex loading conditions.

Most of the research done on characterizing material behavior relies on uniaxial deformation tests. However, when the material is of anisotropic nature, uniaxial deformation tests are not enough to describe their mechanical response when subjected to multiaxial load conditions. Therefore, it is desirable to perform other experimental tests that includes pure shear, biaxial, or equibiaxial deformation states [6], if constitutive material models need to be used to describe the mechanical behavior of polymeric materials. However, there exist major difficulties when performing biaxial tests, starting from the definition of the specimen’s design geometry, clamping and loading devices, technology lab equipment, and sample preparation, to say a few [7,8]. Therefore, one of the goals of this study consists of determining the optimum specimen design geometry to perform equibiaxial tests. We shall start the next section by briefly discussing some of the main previous research related to the identification of the specimen geometry needed to collect equibiaxial experimental data.

### Polymer Biaxial Testing Specimen Geometry

Rivlin and Saunders were the first to use square sheet samples of rubber material to perform biaxial testing in an attempt to produce pure homogeneous deformation [9]. The usage of cross-shaped metallic specimens to investigate biaxial tension in metallic samples was developed by Shiratori and Ikewami [10]. They designed the specimens with a cruciform circular arc with certain dimensions to ensure a region of homogeneous plastic deformation in the middle of the tested samples. Pascoe and Villiers [11] designed flat cruciform specimens to investigate the strain distribution along the principal axes of a metallic flat sheet subjected to equibiaxial loading, and found that this loading condition is the most dangerous deformation state for low-cycle fatigue. In order to perform biaxial tests in samples with different elongations along the strain’s principal axes, Parsons and Pascoe [12] designed a biaxial machine with a closed-loop servo control and used the measured strains or loads as feedback signals. 

A great discussion about the existing biaxial test method, as well as the specimens geometry form and its correlation to Digital Image Techniques and Finite element models, can be found in [13,14], and also in [1,2,5,15,16]. 

Recently, Schubert and co-workers [17] performed equibiaxial tests on magnetorheological elastomers (MREs) subjected up to 10% strain. By using a digital image correlation system, they determined the tangent modules of isotropic and anisotropic MREs. Furthermore, they concluded that the improvement of the machine clamping system, and a larger stiffness of the sliding clamps may affect the experimental results. 

Here, the optimum cruciform geometry that will allow one to subject MRE samples to larger amounts of equibiaxial strain is developed. To validate the proposed specimen geometry, a digital image correlation (DIC) system, Aramis V8, was used. Based on this cruciform geometry, specimens made from polydimethylsiloxane (PDMS, silicone rubber) elastomer reinforced with iron micro- or nanoparticles were subjected to equibiaxial loading and unloading cycles to examine the Mullins effect and residual strain deformations. We shall return to the findings from these studies later on.

The paper is organized as follows. Section 2 of this article focuses on the materials and methods through the following subsections, Section 2.1 and Section 2.2 describe the optimization algorithm and the finite element model used to find the specimen geometry to performed equibiaxial tests with larger samples strains. Section 2.3 summarizes the main characteristics of the reinforced MR materials used to perform the equibiaxial tests by using the optimized cruciform sample geometry. Section 2.4 describes the biaxial test’s experimental equipment. In Section 2.5, a material constitutive model based on a non-Gaussian statistical mechanics model is adapted to predict the equibiaxial stress-stretch deformation state by considering Mullins effects and sample residual deformations. Finally, in Section 3, experimental results collected from the MR material samples reinforced with iron micro- or nanoparticles are compared to numerical results obtained from the constitutive material model, and with those collected from digital image correlation technology.

## 2. Materials and Methods

### 2.1. Cruciform Geometry Optimization

In order to identify the relationship between the strain field and the specimen geometric variables, a design exploration will be performed to determine the influence that strain distributions could have to meet the desired requirements. Here, the geometry parameters have been defined from a tridimensional specimen computational model. Once the model is created and the geometric parameters defined, the next step focuses on creating a sample response surface. As usual, the definition of the design space is made by providing the minimum and maximum values of the input variables. Then, a design of experiment (DOE) is introduced to obtain the design space sampling for each output parameter. Of course, the response surface is an approximation of the response of the system. Its accuracy depends on several factors, such as the complexity of the variations of the output parameters, the number of points in the original DOE, and the choice of the response surface type. In this case, the Kriging scheme will be used to adjust the response surface [18].

Based on this adjusted surface, the Hammersley optimization algorithm is constructed by using the radical inverse function. In this algorithm, any integer *n* can be represented as a sequence of digits $n_{0}n_{1}n_{2},\;\cdots ,\;n_{m}$, by the following equation [19]:

(1) $$n=n_{0}n_{1}n_{2}\cdots n_{m}$$

In general, for a radix *R* representation, the equation

(2) $$n=n_{m}+n_{m-1}\cdot R+\cdots +n_{0}$$

is used. Thus, for a *k*-dimensional search space, the Hammersley points are given by the expression:

(3) $$H_{k}\left(i\right)=\left[i/N,\Phi_{R_{1}}\left(i\right),\Phi_{R_{2}}\left(i\right),\cdots ,\Phi_{R_{k-1}}\left(i\right)\right]$$

where i=0,⋯,N indicate the sample points, and $\Phi_{R}$ given by

(4) $$\Phi_{R}\left(n\right)=n_{m}n_{m-1}n_{m-2}\cdots n_{0}$$

(5) $$\Phi_{R}\left(n\right)=n_{m}\cdot R^{-1}+n_{m-1}\cdot R^{-2}+\cdots n_{0}\cdot R^{-\left(m-1\right)}$$

represents the inverse radical function which generates a fraction in the interval (0, 1) by reversing the order of the digits in Equation (2) about the decimal point.

**Figure 1:**
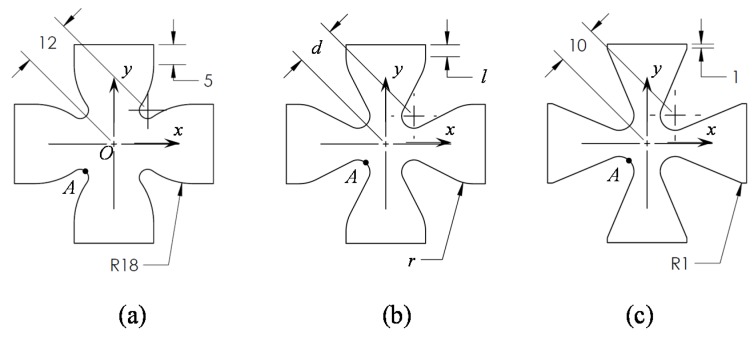
Geometrical shape subjected to surface response analysis in order to obtain the optimal geometry, the extremal values of parameters in (**b**) are shown in (**a**) and (**c**). All dimensions are in millimeters.

Here the variables chosen to perform the Hammersley optimization algorithm of the specimen are the distance *l*, the radius *r* and the distance *d*, as shown in Figure 1. The values of these parameters are assumed to be varying as follows 1 ≤ l ≤ 5, 1 ≤ r ≤ 18, and 10 ≤ d ≤ 12, all dimensions are in millimeters. The objective function consists of minimizing the strain difference between point *A* and the central point *O* of the specimen, shown in Figure 1, in order to have a uniform strain sample state. For illustrative purposes, Figure 1a shows the sample geometry when l=1 mm, r=1 mm, and d=10 mm, while Figure 1c shows the geometry when l=5 mm, r=18 mm and d=12 mm.

### 2.2. Polymer Biaxial Testing Specimen Geometry

**Figure 2:**
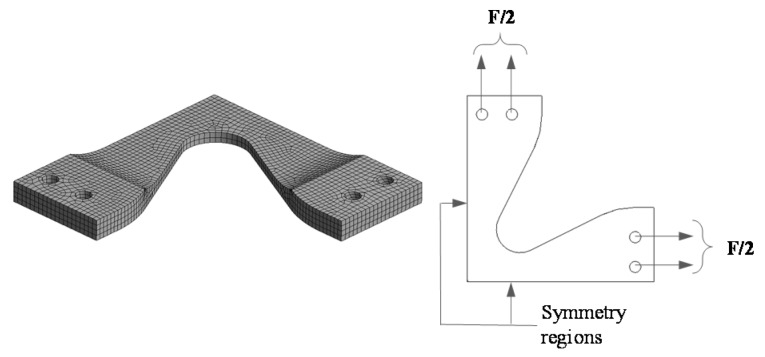
Discretized domain and boundary conditions applied to the finite element model (FEM). Made with 27,168 nodes and 7103 nonlinear elements.

Here, the finite element method (FEM) is used to obtain the sample strain field. In this case, and based on the sample geometry, only a quarter of the specimen is considered, as illustrated in Figure 2. A higher order, three-dimensional, 20-node hexahedron solid element that exhibits quadratic displacement behavior is used. The element is defined by 20 nodes having three degrees of freedom per node: translations in the nodal *x*, *y*, and *z* directions. The FEM model contains 27,168 nodes and 7103 nonlinear elements. The incompressible material constitutive model that describes the mechanical behavior of this element is assumed to be of hyperelastic nature, therefore, this FEM model admits large element deflection and strains [20].

A reduced polynomial form of the material strain energy density introduced by Yeoh is used to obtain the numerical calculations [21]:

(6) $$W=\sum_{i=1}^{N}c_{i0}{\left({\bar{I}}_{1}-3\right)}^{i}+\sum_{k=1}^{N}\frac{1}{d_{k}}{\left(J-1\right)}^{2k}$$

where W is the strain energy per unit volume, ${\bar{I}}_{1}$ represents the first deviatoric strain invariant, μ stands for the initial material shear modulus, $d_{k}$ is the material incompressibility parameter obtained from a data curve fitting process, J represents the determinant of the elastic deformation gradient *F*, N is a material parameter, and $c_{i0}$ represents material constants obtained from a curve fitting data. Here, the initial shear modulus is defined as:

(7) $$\mu =2c_{10}$$

and the initial bulk modulus by:

(8) $$K=\frac{2}{d_{1}}.$$

**Figure 3:**
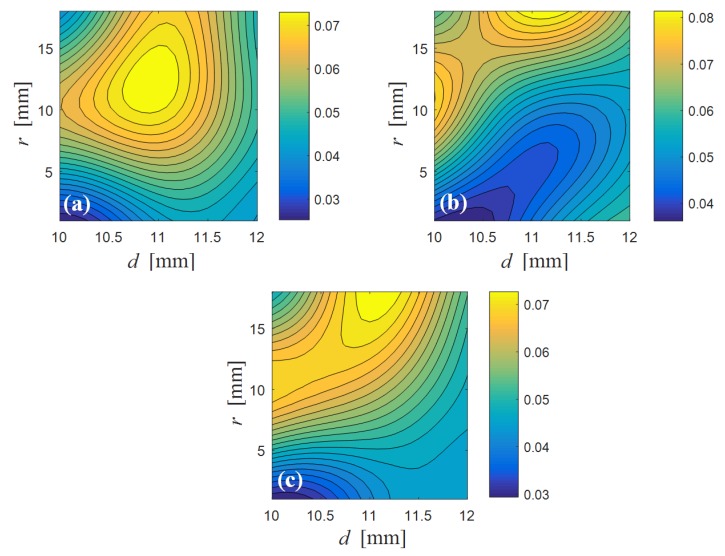
Response surface of the strain difference considering variations of *r* and *l* for three different cases (**a**) l=1.40 mm, (**b**) l=2.64 mm and (**c**) l=1.89 mm.

In this case, N=3 to have a cubic response material function that is a function of the first invariant ${\bar{I}}_{1}$. With these assumptions, the FEM simulations provided the surface response charts shown in Figure 3. These three response surfaces of the strain difference considering variations of *r* and *l* were plotted by assuming: (a) l=1.40 mm, (b) l=2.64 mm, and (c) l=1.89 mm. Here, the response surfaces were obtained from the design of experiments with central composite design in combination with the surface response meta-modeling Kriging algorithm [18].

Table 1 shows the three best candidate points that produce the smallest strain difference between point *A* and *O* obtained from the optimization process. For convenience, the values of the parameters *l*, *d*, and *r* are also included. Additionally, the last two columns contain the values of the objective function Δε that provides the strain difference and the maximum strain obtained in each of the three cases. The selected candidate is number 2 because it produces the highest strain value with a small amount of strain difference.

**Figure 4:**
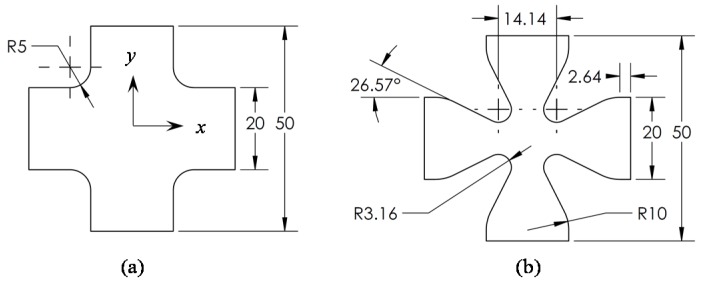
Evolution of the specimen geometry, (**a**) original and (**b**) final shape.

Figure 4 shows the complete evolution of the specimen geometry departing from the original proposed specimen geometry to the optimized geometry, which corresponds to candidate point number 2 in Table 1. Notice that the 50 × 50 mm dimensions remains unchanged, however, as a consequence of this process, a reduction in sample volume from 3047.79 to 1911.54 mm^3^ has been obtained, which represents a volume reduction of about 37%.

**Figure 5:**
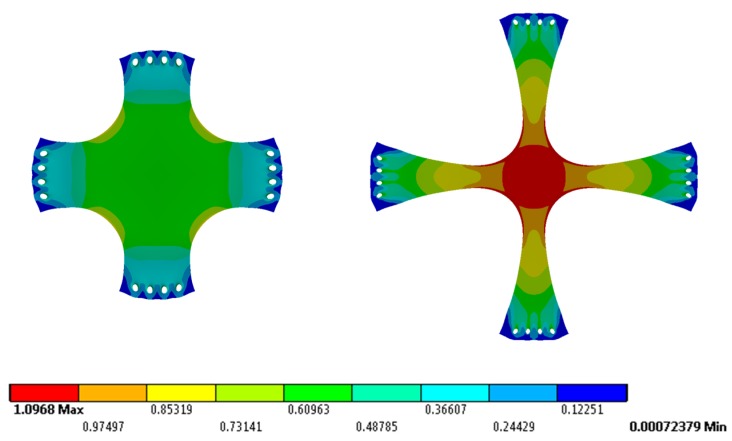
Equivalent strain distribution obtained numerically, in both original and final specimen geometry. In both cases, a tensile load ***F*** of magnitude 1.48 N is applied in *x* and *y* directions.

The equivalent strain fields shown in Figure 5 are numerically obtained when a force ***F*** of magnitude 1.48 N is applied in both the original and optimized specimen dimensions. There is a significant increase in the uniform strain fields in the sample with the optimized geometry that allows performance of equibiaxial experimental tests at larger sample elongations.

**Figure 6:**
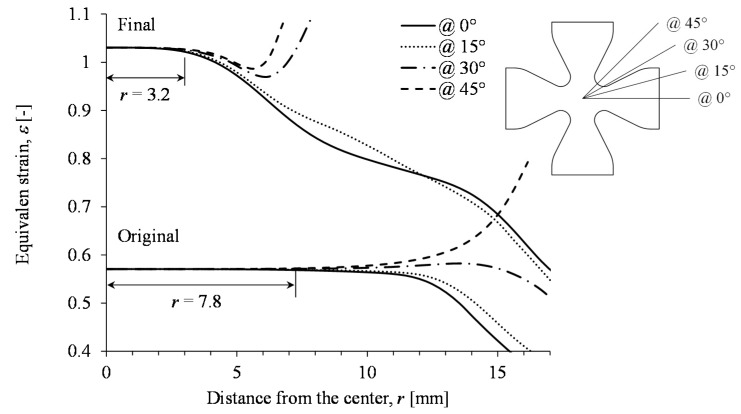
Comparison of equivalent strain values between the original and final specimen geometry along 0°, 15°, 30°, and 45° specimen paths.

In order to evaluate the effectiveness of the proposed geometry, the strain value evolution along four paths are considered. These paths are straigth lines located at 0°, 15°, 30°, and 45°, starting at the center of the sample, as shown in Figure 6. Notice from the curves along these four paths that a zone of equibiaxiality at 0°, 15°, 30°, and 45° is obtained. In this case, the allowed equivalent strain fields’ difference among these paths does not exceed 1%, to guarantee the equibiaxial deformation state. Therefore, the optimized specimen under this load condition could be subjected up to a maximum of 1.03 mm/mm to have a uniform equibiaxial deformation state, which represents an increase of 80% when compared to that of the original sample geometry.

### 2.3. Reinforced Magnetorheological Elastomer Specimens

In this work, silicone rubber (SR) P-85 RTV (shore A hardness: 14) and PE-21 RTV (shore A hardness: 20), silicone oil (SO) with viscosity of 340 cps, and catalyst TP, all purchased from Polisil (México City, México), were used to manufacture the composite materials. 

Two iron spherical particle sizes of magnetic filler were selected: the first one with an average size of 70 nm (CAS 746878 nanoparticles), and the second one with an average size of 2.5 µm (CAS 44890 microparticles), both purchased from Sigma-Aldrich (Monterrey, México) [22]. The iron microparticles are identified by the supplier as carbonyl iron particles, which, according to [23,24,25], are synthesized from thermal decomposition of iron pentacarbonyl (Fe(CO)_5_).

To develop the composite materials, first, the magnetic particles (3.3 vol %) were immersed in an appropriate volume of SO and the mixture was stirred for 3–5 min. Then, 44 g of silicone rubber was added and mixed at room temperature for ~5 min. Before the homogeneous mixture was put into a mold with the geometry of the optimized specimen, the catalyst was added. The curing process was carried out under vacuum conditions to avoid porosity in the specimens at room temperature for 12 h. Two different volumes of silicone oil were used: 4% and 24%, with respect to the volume of SR, to obtain improved MRE material mechanical properties. The samples’ identification nomenclature is shown in Table 2.

**Figure 7:**
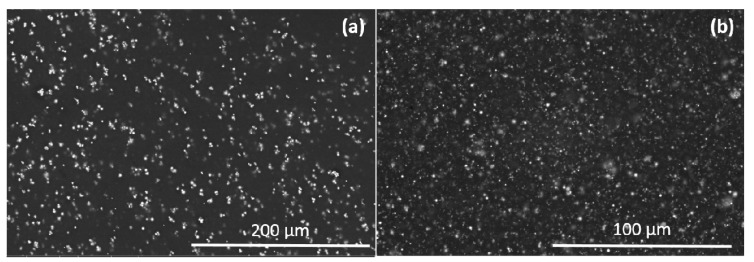
Scanning electron microscopy (SEM) images of iron (**a**) microparticles, and (**b**) nanoparticles embedded into PDMS elastomer matrix material.

To observe the magnetic particles dispersion into the polymeric matrix, scanning electron microscopy (SEM) was performed, using a Quanta 250-FEG FEI (San Luis Potosí, México) operated at 5 kV with an opening of 86 pA. The SEM images of Figure 7 shows a uniform dispersion of iron micro- and nanoparticles and therefore the composite materials are considered to be isotropic [26]. 

### 2.4. Test Rig Configuration

**Figure 8:**
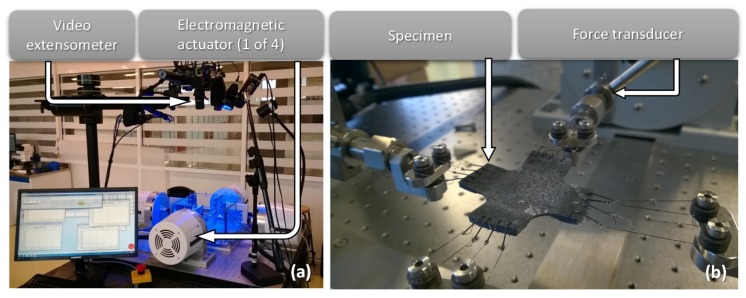
Biaxial test rig configuration, (**a**) video extensometer and linear actuators and (**b**) specimen and force transducers.

For the experimental procedure, an ElectroForce LM1 TestBench (Monterrey, México) was used. The major components of this system consist of four linear actuators (Figure 8a) and load cells attached to the end of the actuators, one in each direction (Figure 8b). This test bench is rated up to 17 N and has a range of displacement of 19 mm. Then, biaxial cyclic loading tests were performed on the MRE samples reinforced with iron micro- or nanoparticles.

### 2.5. Biaxial Stress and Mullin’s Effect

In order to theoretically predict the material response of the composite MRE when subjected to loading and unloading cycles, the rule of mixture material model developed in [27] is adapted to equibiaxial deformations. In this material model, the expression that defines the Cauchy stress-stretch virgin material constitutive equation is given as:

(9) $$T=\left(1-f\right)ℵB+B\frac{2f}{3}\left(A_{1}+\frac{2A_{2}}{3}\left(I_{1}-3\right)\right)-p1$$

where ***T*** is the Cauchy stress tensor, ***B*** is the left Green–Cauchy deformation tensor, $A_{1}$ and $A_{2}$ are material fitting parameters, *f* represents the percentage of particle volumetric fraction contribution, *p* is hydrostatic pressure, 1 represents the identity tensor, and ℵ is a material response function defined by:

(10) $$ℵ=\frac{µ}{3\lambda_{r}}\left[\beta +\frac{1}{N}\left(\frac{1}{\lambda_{r}}-\frac{1}{\beta \left(1-\lambda_{r}^{2}-\frac{2\lambda_{r}}{\beta }\right)}\right)\right],$$

*µ* is the material shear modulus, *N* is the chain number of links, *β* is the inverse of the Langevin function defined as $\beta ={ℒ}^{-1}\left(\lambda_{r}\right)$, *λ_r_* represents the relative chain stretch, $\lambda_{r}=\lambda_{chain}/\lambda_{L}$, with $\lambda_{L}=\sqrt{N}$, $\lambda_{\mathrm{chain}}=\sqrt{I_{1}/3}$, and $I_{1}=\lambda_{1}^{2}+\lambda_{2}^{2}+\lambda_{3}^{2}$. To model the Mullins effect, the following expression is used to describe the stress-softened material behavior [27]:

(11) $$\tau_{j}-\tau_{k}=\left\{\left[\left(1-f\right)ℵ+\frac{2f}{3}\left(A_{1}+\frac{2A_{2}}{3}\left(I_{1i}-3\right)\right)\right]\left(\lambda_{j}^{2}-\lambda_{k}^{2}\right)+\frac{G}{2c}\left[\lambda_{j}f_{i}\left(\lambda_{1},\lambda_{2},\;\lambda_{3}\right)-\;\lambda_{k}f_{k}\left(\lambda_{1},\lambda_{2},\;\lambda_{3}\right)\right]\right\}e^{-b\sqrt{\frac{m}{M}\left(M-m\right)}},\;j\;\neq \;k,\;1,\;2,\;3\;\left(\mathrm{no}\;\mathrm{sum}\right).$$

Here *c* is a constant parameter related to residual strains, *b* is a dimensionless material softening parameter, *m* represents the stretch intensity which is defined for equibiaxial extension as $m=\sqrt{2\lambda^{2}+\lambda^{-8}}$, and $m_{max}=M$ is the amount of maximum strain intensity at the point at which the material is unloaded. The corresponding principal engineering stress ***σ*** could be computed from the expression $\sigma =TF^{-1}$ where $F^{-1}$ represent the inverse of the tensor deformation gradient.

## 3. Results

**Figure 9:**
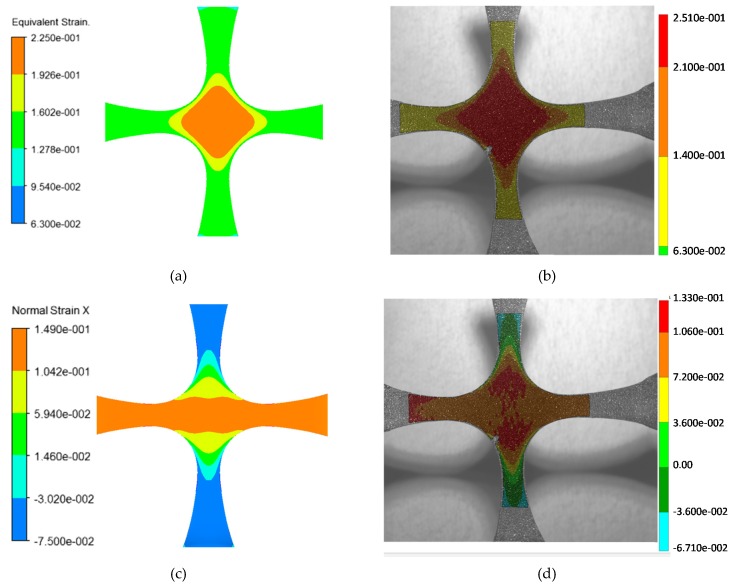
Strain field comparison between the numerically calculated results and those measured experimentally. The applied load is 0.48 N with a preload of 1 N in both *x* and *y* directions. Plots (**a**,**b**) show the equivalent strain contours comparison and plots (**c**,**d**) depict the contours of normal strain in *x* direction.

First, a comparison between the Aramis digital image correlation system with respect to numerical simulations obtained from FEM analysis is addressed. Figure 9 shows that the corresponding contour plots that describe the equivalent and normal strains obtained from both procedures agree well, with a maximum relative error of 5%.

**Figure 10:**
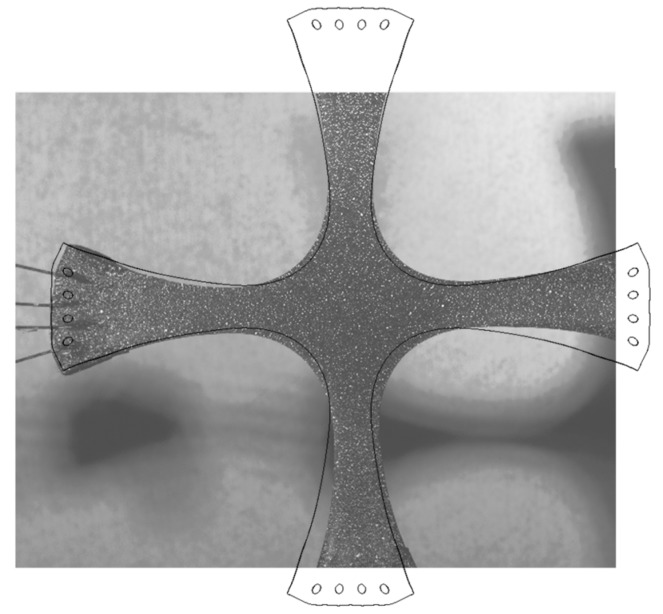
Photograph of the deformed specimen overlaid with the numerically simulated deformed silhouette.

Figure 10 shows the deformed specimen profile obtained from the equibiaxial ElectroForce LM1 TestBench system overlaid with the numerically obtained deformed specimen’s silhouette. During the FEM simulation analysis, two kinds of nonlinearities were considered, one due to the material nonlinearities, and the second related to large deformations to which the higher order, three-dimensional, 20-node solid elements used in the FEM analysis are subjected. This will help us to obtain more accurate results to assess the behavior of the composite material specimens under equibiaxial loadings.

**Figure 11:**
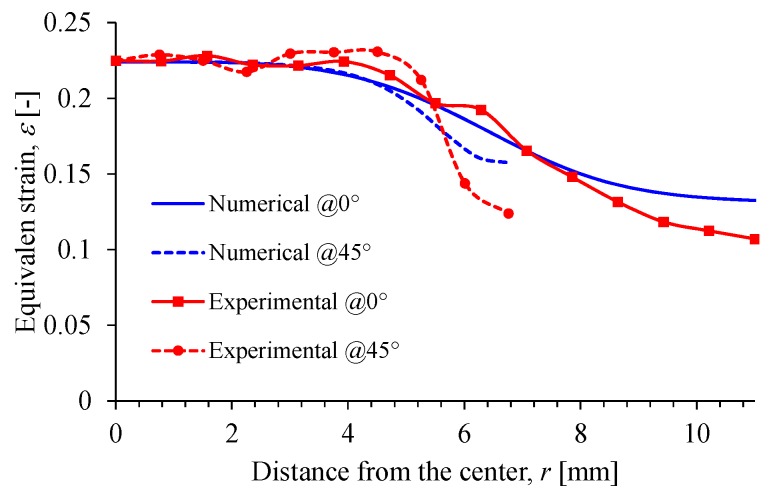
Comparison between experimental and computed strain field. The strain evolution is along two paths at 0° and 45°.

The quantitative comparison between the numerical and experimental values is shown in Figure 11, in which only the strain magnitude along the paths at 0° and 45° were plotted. Based on the FEM numerical results and from the DIC experimental data, it is evident from Figure 11 that both plots agree well in the equibiaxial interval 0≤r≤3.2 mm where the maximum relative error between both procedures does not exceed of 5%.

**Figure 12:**
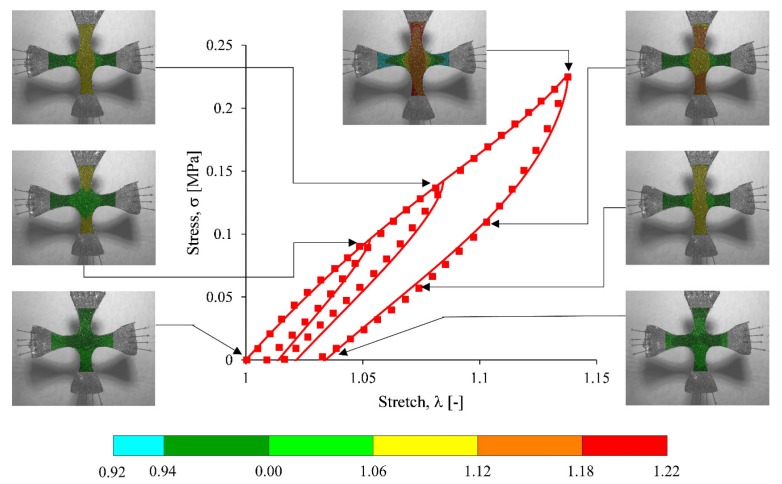
Pictures of the NS24 sample during the loading and unloading equibiaxial cyclic test, the contours illustrate experimental values of stretch λ in *y* direction. These pictures are overlapped on their corresponding NS24 equibiaxial stress-stretch plot.

**Figure 13:**
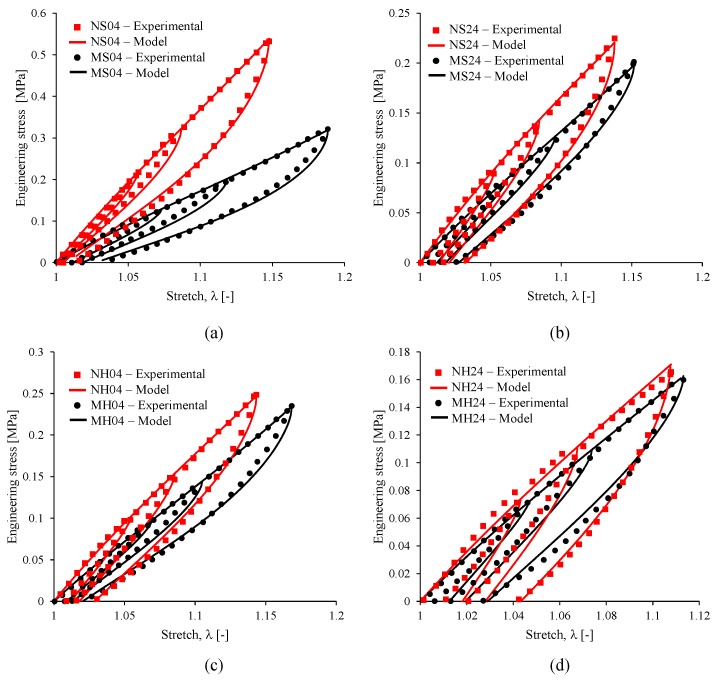
Experimental data of loading and unloading cycles for magnetorheological elastomers with 3.3 vol % of iron nanoparticles. The experimental measurements are indicated in black dots and red squares, while the solid lines are mathematical model predictions obtained from Equation (11).

Next, loading and unloading equibiaxial cyclic tests were performed with the reinforced MRE material specimens using the optical technique described above. Figure 12 shows pictures of the NS24 sample during the cyclic test, overlaid on their corresponding NS24 equibiaxial stress-stretch plot. Experimental data curves obtained from the reinforced elastomers, listed in Table 2, are shown in Figure 13, in which the addition of micro and nanoparticles to the silicone matrix tends to increase the material stiffness, as shown in Table 3. Notice that theoretical predictions and experimental data tend to have good agreement. Here, black dots indicate experimental data, while the solid lines are theoretical predictions computed from Equation (11). The material shear modulus *µ*, the chain number of links *N*, the energy density parameters *A*_1_ and *A*_2_, the stress softening and residual stress parameters *b* and *c*, respectively, used to fit experimental data in Equation (1) are summarized in Table 3. From Table 3, it is concluded that the addition of iron nanoparticles into the elastomeric matrix material increases their stiffness. Furthermore, stress softening and permanent set effects are appropriately captured by the material model (11), when the corresponding material parameters *b* and *c* are fitted with the values shown in Table 3.

## 4. Conclusions

An optimum cruciform specimen geometry has been obtained by combining optimization algorithms and finite element simulations to perform equibiaxial tests at increasing homogeneous strain values. Comparison of the optimum specimen geometry when subjected to equibiaxial deformations via FEM simulations with respect to experimental data collected from DIC measurements showed that the maximum relative error attained does not exceed 5%. In fact, when a force F=1.48 N is applied to the optimized specimen, experimental results show that these specimens can be subjected to larger equibiaxial deformations and still have uniform strain distribution fields to collect accurate experimental data, as illustrated in Figure 11. To further assess the applicability of the obtained cruciform geometry, specimens produced from MRE reinforced with iron micro- or nanoparticles were subjected to equibiaxial loading and unloading cycles to examine the Mullins effect and residual strain deformations. From theoretical predictions and experimental results, it is concluded that the addition of iron nanoparticles increases the material shear modulus when compared to those specimens reinforced with iron microparticles. However, this increasing value in the shear modulus observed in the reinforced PDMS polymer with iron nanoparticles could not be possible if other nanoparticles are used as fillers, unless some care is taken during the material’s preparation, with regards to properties such as (1) good dispersion, (2) morphological form of the nanoparticles, (3) maximum accessible shear forces during the sample mixing preparation, (4) percentage of added nanoparticles, to say a few [28,29,30,31].

Finally, the findings of this article suggest that embedding iron micro and nanoparticles in a non-magnetizable elastomer matrix is of practical interest, since this composite material can vary the mechanical stiffness of the matrix when under the influence of magnetic fields. For instance, these magnetorheological elastomers can be used in prosthetic devices and in the design and manufacturing of smaller and lighter components, such as fuel injectors and fuel pumps, among others [32,33].

## Figures and Tables

**Table 1:** Candidate points obtained from the optimization process.

Candidate Point	*l* (mm)	*d* (mm)	*r* (mm)	Δ*ε* (-)	*ε_max_* (-)
1	1.40	10.17	2.59	0.033	0.88
2	2.64	10.00	10.05	0.054	0.97
3	1.89	10.01	7.38	0.067	0.97

**Table 2:** Nomenclature definition of magnetorheological elastomer (MRE) samples.

Nomenclature	Filler	Shore a Hardness	Silicon Oil Volume %
MS04	Microparticles	14 (Soft)	04
NS04	Nanoparticles	14 (Soft)	04
MS24	Microparticles	14 (Soft)	24
NS24	Nanoparticles	14 (Soft)	24
MH04	Microparticles	20 (Hard)	04
NH04	Nanoparticles	20 (Hard)	04
MH24	Microparticles	20 (Hard)	24
NH24	Nanoparticles	20 (Hard)	24

**Table 3:** Material constants used to fit experimental data.

Sample	*μ* (MPa)	*N* (-)	*A*_1_ (MPa)	*A*_2_ (MPa)	*b* (-)	*c* (MPa)	*f* (-)	Permanent Set
MS04	1.94	2.62	−58.27	0	1.18	14.28	0.033	1.032
NS04	3.61	2.29	−102.70	0	1.20	39.48	0.033	1.014
MS24	1.44	2.41	−42.58	0	0.76	11.05	0.033	1.024
NS24	2.24	3.76	−72.40	0	1.32	9.59	0.033	1.031
MH04	1.52	2.28	−44.90	0	0.88	18.01	0.033	1.020
NH04	2.12	2.93	−65.31	0	1.01	10.43	0.033	1.029
MH24	1.53	2.51	−44.93	0	1.01	7.00	0.033	1.026
NH24	2.14	2.99	−67.87	0	1.68	4.5	0.033	1.042
